# Rare acute exacerbation of lewis-sumner syndrome mimicking stroke: a case report

**DOI:** 10.1007/s10072-025-08609-6

**Published:** 2026-01-06

**Authors:** S. Mambriani, R. Brusa, L. Politini, A. C. Prelle

**Affiliations:** 1 Università Degli Studi Di Milano, Milano, Italy; 2 Neurologia-Stroke Unit, Dipartimento Di Neuroscienze, ASST Ovest MI, Sede Di Legnano, Italy

Lewis-Sumner syndrome (LSS), or multifocal acquired demyelinating sensory and motor neuropathy (MADSAM), is a rare variant of chronic inflammatory demyelinating polyneuropathy (CIDP) characterized by persistent conduction blocks in motor and sensory nerves. LSS accounts for 5–10% of CIDP cases, with a prevalence of 1–9/1,000,000 [1]. While the exact cause of this condition is not fully understood, there are several factors believed to contribute to its development, in particular autoimmune disorders and viral infections. We describe an acute exacerbation of LSS during an infection of Influenza A virus, supporting hypothesis of an immune-mediated etiology.

We present the case of a 63-year-old man affected by LSS. In 2017, he has developed a deficit in dorsiflexion of the left foot and paresthesia in the hands, with a prevalence of the left one; in 2019, following a clinical worsening, he was hospitalized and the lumbar puncture revealed albumino-cytological dissociation (white cells: 2/uL, protein: 80 mg/dL) in the Cerebrospinal fluid (CSF). Nerve conduction studies and electromyography showed a multifocal demyelinating polyneuropathy with motor and sensitive conduction blocks affecting both upper and lower limb with an asymmetric pattern. Antiganglioside IgG and IgM antibodies, like anti-GQ1b, antiGM1, anti-GD1a, anti-GD1b and anti-GM2 were negative. According to the European guidelines [2], the diagnosis of multifocal CIDP was made.

The clinical picture remained almost unchanged with the initial corticosteroids therapy. He responded to intravenous immunoglobulins (IVIg), albeit with an early end-of-dose effect after three weeks. In the following months, he had good control of the symptoms with the weekly administration of intravenous immunoglobulins (30–35 g per week), with an inflammatory neuropathy cause and treatment (INCAT) disability score of 2 point (mild weakness of the interosseous and finger extensors, deficit of dorsiflexion of the left foot).

In January 2025, during a routine IVIg infusion, he developed sudden severe left brachio-crural hemiparesis, prompting urgent neurological evaluation and imaging studies for suspected acute cerebrovascular event. Brain CT and intracranial CT angiography were unremarkable. Blood tests were normal except for a modest increase in creatine kinase (a known finding). Given his history of hypertension and clinical presentation, the patient underwent intravenous thrombolysis with Alteplase without neurological benefit. Within 24 h, he developed fever, significant desaturation requiring continuous positive airway pressure (CPAP) ventilation, tetraparesis, areflexia, dysphagia, dysarthria, and subsequently diplopia. Due to progressive respiratory deterioration, the patient was intubated and admitted to the Intensive Care Unit with the INCAT disability score of 10. He presented significant involvement of the cranial nerves, with diplopia in all directions of gaze, moderate deficit of both the superior and inferior component of the VII right cranial nerve, dysphagia with difficulty in managing secretions. Suspecting an acute exacerbation of his known chronic demyelinating disease, a five-day course of IVIg (2 g/kg) was initiated, leading to rapid improvement in neurological status and respiratory function immediately after the infusions. An electromyography was then performed, which documented a picture of motor demyelinating polyneuropathy with conduction blocks; no decrease in the amplitude of compound muscle action potentials to low-frequency repetitive stimulation (Table 1).

**Table 1:** Motor and sensory nerve conduction studies

MNCS						
NERVE	Latency (ms)	Amplitude (mV)	Conduction Velocity (m/s)	F-M latency (ms)	Distance (mm)	
LEFT PERONEUS						
ANKLE-EDB	5.28	5.9			80	
BL. KNEE-ANKLE	14.7	6	34		320	
LEFT TIBIALIS						
ANKLE-ABD HAL	5.8	8		11.1	120	
KNEE-ANKLE	19.3	3.4	34.4		465	
RIGHT TIBIALIS						
ANKLE-ABD HAL	6.37	6.4			120	
KNEE-ANKLE	17.7	1.31	39.7		450	
RIGHT ULNARIS						
WRIST-ADM	2.66	10		24.5	60	
BL. ELBOW-WRIST	7.94	3.1	42.6		225	
AB. ELBOW-BL-ELBOW	10.3	2.9	59.3		140	
SNCS						
NERVE	Peak Latency (ms)	Amplitude (mV)	Conduction Velocity (m/s)	Distance (mm)	Int stimolus mA	
RIGHT ULNARIS		uV Pos2-neg	uVPo1-neg			
WRIST-DIGIT V	2.27	6.7	4.3	52.9	120	21.4

Right peroneal nerve: increased distal latency, reduced CMAP amplitude and MCV. Right ulnar nerve: normal distal latency, > 50% amplitude drop forearm–wrist, preserved conduction velocity. Marked CMAP temporal dispersion after proximal stimulation (except left superficial peroneal)

Right ulnar SNAP: reduced amplitude

Absent F-waves: left peroneal and right ulnar nerves

*MNCS* motor nerve conduction study, *SNCS* sensory nerve conduction study, *EDB* extensor digitorum brevis, *BL* below, *AB* above, *ABD HAL* abductor hallucis, *ADM* abductor digiti minimi

Since the patient initially had a fever, an analysis of bronchoalveolar lavage fluid showed positive result for influenza A virus (H1N1). The exam on CFS was normal. As a systemic complication, the patient progressively developed anemia with iron and folic acid deficiency and a direct Coombs test positive.

We concluded for anemia secondary to a systemic inflammatory state with modest parainfectious hemolysis, for which only supplemental iron and folic acid therapy was necessary. The patient recovered completely and at subsequent visits, he showed clinical stability (INCAT disability score of 2) and he didn’t present new neurological symptoms.

This case highlights an acute LSS relapse, associated with Influenza A infection, that mimicked a stroke. Various infectious agents, like Influenza virus or hepatitis C virus (HCV), have been reported as relapse tiggers for CIDP. There are some cases secondary also to COVID-19 infection [3]. The only comparable case [4] described a patient with LSS with hyperacute relapse mimicking stroke and with H1N1 infection. He was treated only with oseltamivir with significant clinical improvement. In this case, antiviral therapy may have been sufficient, considering that some viruses act by directly inhibiting sodium and potassium channels, thus altering the propagation of nerve impulses. In our case, we hypothesize that a systemic pro-inflammatory state triggered by the Influenza A virus has determined a significant activation of autoimmunity which was subsequently suppressed by IVIg.

Cranial nerve involvement occurs more frequently in LSS than in CIDP, across both typical and atypical variants. Most LSS relapses are not infection-related; consequently, cranial nerve deficits in LSS do not appear to be highly frequently linked to an infectious trigger, but further studies are needed to demonstrate this.

Fever-related relapses have also been described in CIDP, characterized by abrupt neurological symptoms onset during pyrexia and rapid remission after defervescence; the symptoms were stereotypical in every relapse. The mechanism is uncertain, but inflammatory cytokines (IL-1β, IL-2, TNF-α) may impair conduction at proximal nerve sites where the blood–nerve barrier (BNB) is disrupted [5] by chronic demyelination.

In LSS, CSF protein elevation- that correlates with lesions of proximal nerve segments- is less frequent [6]. It’s hypothesized that there’s a breakdown of the BNB mediated by local activation of T cells, macrophages, celladhesion molecules, cytokines, etc. that cause then the characteristic conduction blocks [7].

Our case illustrates a hyperacute relapse immediately after IVIg administration and rapid recovery with the same therapy. We hypothesize an immune-mediated flare, with systemic inflammation, triggered by Influenza A. Unfortunately, cytokine levels were not measured, limiting our conclusions. This was the first such relapse in this patient, and a clear link between fever and exacerbation remains uncertain. Further research into LSS and CIDP is needed to clarify relapse mechanisms and recurrence risk.
